# Complete Genome Sequences of Six Copper-Resistant *Xanthomonas* Strains Causing Bacterial Spot of Solaneous Plants, Belonging to *X. gardneri*, *X. euvesicatoria*, and *X. vesicatoria*, Using Long-Read Technology

**DOI:** 10.1128/genomeA.01693-16

**Published:** 2017-02-23

**Authors:** Damien Richard, Claudine Boyer, Pierre Lefeuvre, Blanca I. Canteros, Shyam Beni-Madhu, Perrine Portier, Olivier Pruvost

**Affiliations:** a CIRAD, UMR PVBMT, St.-Pierre, Réunion, France; b ANSES, Plant Health Laboratory, St.-Pierre, Réunion, France; c Université de la Réunion, UMR PVBMT, St.-Denis, Réunion, France; d INTA, Estación Experimental Agropecuaria Bella Vista, Buenos Aires, Argentina; e FAREI, Le Réduit, Mauritius; f INRA, UMR1345 IRHS Institut de Recherche en Horticulture et Semences, Beaucouzé, France

## Abstract

*Xanthomonas vesicatoria*, *Xanthomonas euvesicatoria*, and *Xanthomonas gardneri* cause bacterial spot disease. Copper has been applied since the 1920s as part of integrated management programs. The first copper-resistant strains were reported some decades later. Here, we fully sequenced six *Xanthomonas* strains pathogenic to tomato and/or pepper and having a copper-resistant phenotype.

## GENOME ANNOUNCEMENT

Bacterial spot disease causes yield loss and impairs fruit quality in most tomato- and pepper-producing areas. The disease is caused by several distinct *Xanthomonas* species: *X. vesicatoria*, *X. euvesicatoria*, *X. gardneri*, and *X. perforans*, with *X. perforans* being recently reclassified as *X. euvesicatoria* (1). These phylogenetically distant species display similar symptoms and host range (2). Tomato (*Lycopersicon esculentum*), sweet pepper (*Capsicum annuum*), and chili pepper (*Capsicum frutescens*) are the main natural host species. The large-scale spread of the pathogens occurs through contaminated seeds, requiring strict disease management strategies to be imposed. A research effort is being made on biocontrol strategies, such as bacteriophage use, because resistant cultivars are scarce and rapidly overcome, and bacterial resistance to antimicrobial compounds is often reported. For example, widely used on pepper since the 1920s in the United States (3), a failure of copper sprays to control bacterial spot disease was first observed in 1968 (4), and the first associated genetic determinant (i.e., a transmissible plasmid) was reported more than 20 years later (2, 5).

In order to obtain a better understanding of copper resistance determinants and their spread among xanthomonads, we sequenced six tomato and pepper pathogen strains displaying a copper-resistant phenotype: *X. euvesicatoria* LMG930 and LH3, *X. vesicatoria* LMG911 and LM159, and *X. gardneri* JS749-3 and ICMP7383.

The long-read PacBio RSII technology was used to fully sequence the six strains, using one single-molecule real-time (SMRT) cell for each strain. Assembly of the raw reads was then performed using a SMRT Analysis HGAP version 2.3 protocol, and circularization of the contigs was done using a combination of the Minimus assembler (6) and the SMRT Analysis resequencing version 1 protocol. Assembly of transcription activator-like genes was improved using a custom version of the method previously described (7).

We obtained six closed chromosomes with sizes ranging from 4,969,893 bp to 5,313,102 bp, along with two to four closed plasmids per strain (31,328 bp to 222,061 bp) and uncircularized contigs. Average nucleotide identity (ANI) scores with type strains were used to confirm species assignation (Table 1). The six strains possessed the previously described copper resistance gene system *copLAB* (8) carried on a conjugative plasmid. Interestingly, the plasmid was very well conserved among five strains (dating from 1955 to 2010), while the other (LMG930) had no similarities apart from the copper resistance gene cluster. On five strains, this plasmid also comprised the *cusAB-smmD*, *czcABCD*, and *arsBHCR* resistance gene cluster. LH3 solely lacked *arsBHCR*. Evaluation of the minimum inhibitory concentrations was achieved on casitone yeast extract glycerol (CYE) medium, as previously reported (9, 10): these were zinc chloride, 16 to 32 mg/liter; copper sulfate, 128 to 256 mg/liter; sodium arsenite, 16 mg/liter (LH3), 128 to 512 mg/liter (other strains); cadmium sulfate, 6.4 to 12.8 mg/liter; and cobalt chloride, 16 to 32 mg/liter.

**TABLE 1:** Characteristics of the six sequenced *Xanthomonas* strains^a^

Strain	Other no.	Species	Copper resistance location	Country of isolation	Yr of isolation	Host	Chromosome size (bp)	G+C content (%)	ANI score (%)^b^	Accession no.
LMG930		*X. euvesicatoria*	Plasmid	United States	1969	Pepper	5,079,107	64.7	99.98	CP018463 to CP018467
LMG911	CFBP2537	*X. vesicatoria*	Plasmid	New Zealand	1955	Tomato	5,110,163	64.3	99.95	CP018725 to CP018727
LM159	Bv-5-4a (INTA)	*X. vesicatoria*	Plasmid	Argentina	1987	Pepper	5,086,726	64.2	99.88	CP018468 to CP018471
LH3	Xv5 (FAREI), CFBP7993	*X. euvesicatoria^c^*	Plasmid	Mauritius	2010	Tomato	4,969,893	64.9	98.68 (99.98)	CP018472 to CP018476
JS749-3		*X. gardneri*	Plasmid	Réunion	1997	Tomato	5,158,913	63.7	99.91	CP018728 to CP018730
ICMP7383	CFBP7999	*X. gardneri*	Plasmid	New Zealand	1980	Tomato	5,313,102	63.5	98.29	CP018731 to CP018734

a LMG strains are from the Belgian Coordinated Collections of Microorganisms (BCCM/LMG), University of Ghent, Belgium. ICMP, International Collection of Microorganisms from Plants, Landcare Research, Auckland, New Zealand; INTA, Instituto Nacional de Tecnología Agropecuaria; FAREI, Food and Agricultural Research and Extension Institute; CFBP, Collection Française de Bactéries Associées aux Plantes.

b ANI scores were calculated using type strains of each species: LMG27970 for *X. euvesicatoria*; ATCC 35937 for *X. vesicatoria*; and ATCC 19865 for *X. gardneri*. Value in parentheses is for the type strain of *X. perforans* (CFBP7293).

c Synonym of *X. perforans*.

The very high similarity of the plasmids hosting the copper resistance adaptive trait among four species pathogenic to solaneous plants suggests pervasive events of horizontal gene transfer at the niche level.

### Accession number(s).

The six closed genome sequences have been deposited at GenBank. The genome accession numbers are shown in Table 1.

## References

[1] Constantin EC, Cleenwerck I, Maes M, Baeyen S, Van Malderghem C, De Vos P, Cottyn B 2016 Genetic characterization of strains named as *Xanthomonas axonopodis* pv. dieffenbachiae leads to a taxonomic revision of the *X. axonopodis* species complex. Plant Pathol 65:792–806. doi:10.1111/ppa.12461.

[2] Potnis N, Timilsina S, Strayer A, Shantharaj D, Barak JD, Paret ML, Vallad GE, Jones JB 2015 Bacterial spot of tomato and pepper: diverse *Xanthomonas* species with a wide variety of virulence factors posing a worldwide challenge. Mol Plant Pathol 16:907–920. doi:10.1111/mpp.12244. 25649754 PMC6638463

[3] Higgins BB 1922 The bacterial spot of pepper. Phytopathology 12:501–516.

[4] Marco GM, Stall R 1983 Control of bacterial spot of pepper initiated by strains of *Xanthomonas campestris* pv. vesicatoria that differ in sensitivity to copper. Plant Dis 67:779–781. doi:10.1094/PD-67-779.

[5] Stall RE, Loschke DC, Jones JB 1986 Linkage of copper resistance and avirulence loci on a self-transmissible plasmid in *Xanthomonas campestris* pv. vesicatoria. Phytopathology 76:240–243. doi:10.1094/Phyto-76-240.

[6] Sommer DD, Delcher AL, Salzberg SL, Pop M 2007 Minimus: a fast, lightweight genome assembler. BMC Bioinformatics 8:64. doi:10.1186/1471-2105-8-64. 17324286 PMC1821043

[7] Booher NJ, Carpenter SC, Sebra RP, Wang L, Salzberg SL, Leach JE, Bogdanove AJ 2015 Single molecule real-time sequencing of *Xanthomonas oryzae* genomes reveals a dynamic structure and complex TAL (transcription activator-like) effector gene relationships. Microb Genom 1. doi:10.1099/mgen.0.000032. PMC4853030 27148456

[8] Behlau F, Canteros BI, Minsavage GV, Jones JB, Graham JH 2011 Molecular characterization of copper resistance genes from *Xanthomonas citri* subsp. *citri* and *Xanthomonas alfalfae* subsp. *citrumelonis*. Appl Environ Microbiol 77:4089–4096. doi:10.1128/AEM.03043-10. 21515725 PMC3131652

[9] Zevenhuizen LPTM, Dolfing J, Eshuis EJ, Scholten-Koerselman IJ 1979 Inhibitory effects of copper on bacteria related to the free ion concentration. Microb Ecol 5:139–146. doi:10.1007/BF02010505. 24232421

[10] Pruvost O, Couteau A, Perrier X, Luisetti J 1998 Phenotypic diversity of *Xanthomonas* sp.* mangiferaeindicae*. J Appl Microbiol 84:115–124. doi:10.1046/j.1365-2672.1997.00328.x. 15244066

